# Highly Active and Stable Pt–Pd Alloy Catalysts Synthesized by Room‐Temperature Electron Reduction for Oxygen Reduction Reaction

**DOI:** 10.1002/advs.201600486

**Published:** 2017-01-20

**Authors:** Wei Wang, Zongyuan Wang, Jiajun Wang, Chuan‐Jian Zhong, Chang‐Jun Liu

**Affiliations:** ^1^ Collaborative Innovation Center of Chemical Science and EngineeringSchool of Chemical Engineering and TechnologyTianjin UniversityTianjin300072P. R. China; ^2^ Department of ChemistryState University of New York at BinghamtonBinghamtonNY13902USA

**Keywords:** alloy, electron reduction, oxygen reduction reaction, palladium, platinum

## Abstract

Carbon‐supported platinum (Pt) and palladium (Pd) alloy catalyst has become a promising alternative electrocatalyst for oxygen reduction reaction (ORR) in proton exchange membrane fuel cells. In this work, the synthesis of highly active and stable carbon‐supported Pt–Pd alloy catalysts is reported with a room‐temperature electron reduction method. The alloy nanoparticles thus prepared show a particle size around 2.6 nm and a core–shell structure with Pt as the shell. With this structure, the breaking of O–O bands and desorption of OH are both promoted in electrocatalysis of ORR. In comparison with the commercial Pt/C catalyst prepared by conventional method, the mass activity of the Pt–Pd/C catalyst for ORR is shown to increase by a factor of ≈4. After 10 000‐cycle durability test, the Pt–Pd/C catalyst is shown to retain 96.5% of the mass activity, which is much more stable than that of the commercial Pt/C catalyst.

## 1 Introduction

Due to the high power density, the high efficiency and clean emissions, proton exchange membrane fuel cells (PEMFCs) have attracted a great attention as a promising alternative to fossil fuel‐based thermal combustion engine for vehicles.^1^ At present, carbon‐supported platinum (Pt) is still the widely used cathode catalyst in PEMFCs.^2^ However, for applications of the Pt/C catalyst, there are still some critical issues to be solved, including sluggish kinetics of the oxygen reduction reaction (ORR), the poor stability, and the expensive price.^3^ Therefore, a number of strategies have been proposed for enhancing the electrocatalytic properties and reducing the cost of Pt‐based catalysts, such as improving the carbon support,^4^ changing the morphologies of Pt nanoparticles,^5^ and synthesizing Pt–M alloy catalysts.^6^

Among these strategies, the approach to fabricate the Pt–M alloy catalysts has achieved significant progresses due to many advantages.^7^ By testing Pt alloy films with iron (Fe), cobalt (Co), and nickel (Ni), Nørskov and his co‐workers^8^ found that Pt_3_Co alloy film showed much higher ORR area activity than that of pure Pt. Stamenkovic and his co‐workers^9^ fabricated PtFe alloy nanoparticles and found that the mass activity of Pt_3_Fe nanoparticles was three times higher than that of Pt NPs. Although Pt–M alloys with transition metals have shown improved ORR activity, the low redox potentials of these transition metals (Co −0.28 V, Fe −0.44 V, and Ni −0.25 V) and their dissolution during PEMFC operation remain a significant challenge for a large scale application.^10^ To overcome this challenge, Pt alloys with other noble metals, especially with Pd, have attracted many attentions.^11^ Choi and his co‐workers synthesized palladium–platinum core–shell icosahedra catalyst. They found that the ORR activity of this alloy catalyst was 7.8 times greater compared with the commercial Pt/C catalyst.[[qv: 11a]] Schmidt and his co‐workers[[qv: 11b]] prepared Pt−Pd hydrogels by adding NaBH_4_ into the mixed precursor solution. They found that the activity of the Pt−Pd hydrogel was 4.8 times higher than that of the commercial Pt/C catalyst. After 10 000‐cycle durability test, the Pt−Pd hydrogel was shown to retain 88% of the mass activity. Therefore, Pt−Pd alloy catalysts have shown outstanding ORR activity and stability compared with the commercial Pt/C catalyst.

According to the theoretical and experimental studies, when alloyed with Pd, the d‐band center energy of Pt is decreased so that the activity for ORR could be enhanced by a factor of 1.5–3.^12^ Since the lattice constants and chemical reactivity of Pt and Pd are very similar, they can be readily prepared as alloy or core–shell nanocrystals through chemical or electrical methods.^13^ In addition, Pd is a good candidate to help reduce the high cost of the Pt/C catalyst because the current price of Pd is only half of that of Pt. At last, the Pt–Pd alloy catalyst also shows better catalytic durability, since the addition of Pd could prevent the corrosion of Pt by sacrificing Pd or up‐shifting the dissolution potential of Pt.^14^

To date, the Pt–Pd alloy catalysts are typically prepared by impregnation and deposition methods.^15^ For impregnation method, metal salt precursors are usually reduced by chemical reduction in a solution mixed with the support.[[qv: 15a]] This method is simple, fast, and convenient, but sometimes the size and the composition of alloy nanoparticles could not be controlled precisely.^16^ For deposition method, Pt atoms are usually chemically or electrically deposited as ultrathin shells on Pd NPs to form core–shell structure.[[qv: 15b]] With this method, the composition and the structure of alloy NPs could be adjusted as required. However, the large scale synthesis and further applications of this method may be inhibited due to the complexity of controlling the high temperature and slow injection.^17^ Therefore, it is important to develop a better method for simple and fast synthesis of Pt–Pd alloy catalysts with well‐defined composition and structure.

Herein, we report the synthesis of highly active and stable carbon‐supported Pt–Pd alloy catalysts for PEMFCs cathode using room‐temperature electron reduction with argon glow discharge as electron source.^18^ Glow discharge is well known as a conventional cold plasma phenomenon with highly energetic electrons, with which the reduced metal NP shows small size, strong interaction with the support and high catalytic property.^19^ This method needs neither chemical reducing agents nor protective chemical nor dispersing agents. In addition, when the precursor is a mixture of different metal salts, the electron reduction can also lead to the formation of alloy NPs with small particle size. With this method, we have made Au–Pd alloy catalysts with high catalytic activity.^20^ In this work, we demonstrate the use of the room‐temperature electron reduction for the preparation of Pt–Pd alloy NPs with very small size supported on carbon. The size of obtained alloy NPs is only 2.6 nm and the alloy NPs feature a core–shell structure. The mass activity (MA) of the Pt–Pd/C catalyst is four times greater than that of the commercial Pt/C catalyst (Alfa Aesar). After 10 000 cycles in a typical durability test protocol, the ORR stability of our Pt–Pd/C is 96.5%, which is much higher than that of the commercial Pt/C catalyst, demonstrating the carbon‐supported Pt–Pd alloy catalyst thus prepared as a promising choice for the ORR with high activity and stability.

## 2 Results

### 2.1 Characterization

**Figure 1:**
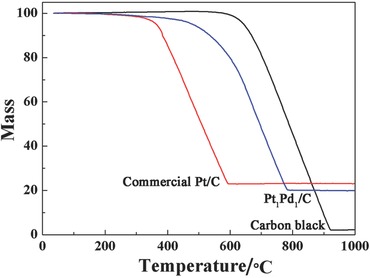
TGA curves of commercial Pt/C and Pt_1_Pd_1_/C catalysts and carbon black.

The metal loading of commercial Pt/C and Pt_1_Pd_1_/C catalysts was determined by the thermogravimetric analysis (TGA) analysis, as shown in **Figure** **1**. It can be found that the pure carbon black is decomposed completely at 1000 °C, suggesting high purity of the support. From the TGA results, the Pt loading of the commercial Pt/C catalyst is 23%, while the metal loading of the Pt_1_Pd_1_/C catalyst is 19.9%. The TGA results of other catalysts are shown in Figure S1 (Supporting Information). The metal loadings of Pt_1_Pd_2_/C, Pt_1_Pd_4_/C, and Pt_1_/C catalysts are 14.9%, 12.5%, and 10.0%, respectively.

**Figure 2:**
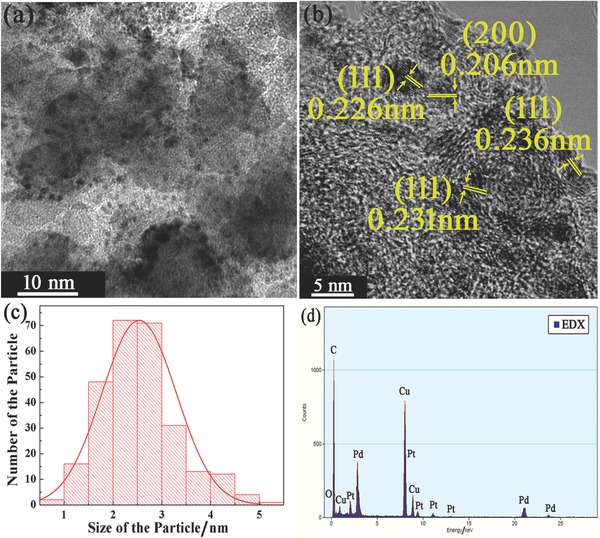
TEM results of the Pt_1_Pd_1_/C catalyst. a,b) TEM images, c) the corresponding particle size distribution, and d) the EDS spectrum.

**Figure** **2** shows the transmission electron microscope (TEM) results of the Pt_1_Pd_1_/C catalyst. From Figure 2a, we can find that the Pt–Pd alloy NPs dispersed well on the carbon, while the even size of these NPs is 2.6 nm according to the particle size distribution in Figure 2c. Such small size suggests that the electron reduction can control the size of alloy NPs effectively. In Figure 2b, the lattice fringes detected are attributed to mainly (111) facet, which is the characteristic of metal NPs reduced by electron reduction method.^18^ The enlarged and clear lattice fringes of metal NPs in Figure 2b are shown in Figure S2 (Supporting Information). From energy‐dispersive X‐ray spectrometer (EDS) spectrum shown in Figure 2d, besides the C element, Pt and Pd elements are also detected, while the Cu peaks are due to the use of copper grid. According to EDS results, the Pt/Pd weight ratio is almost 1:1, which is the same as the feeding mass ratio. Therefore, the Pt loading of the Pt_1_Pd_1_/C catalyst is about 10%. The TEM images of Pt_1_Pd_2_/C and Pt_1_Pd_4_/C catalysts are shown in Figures S3 and S4, respectively, in the Supporting Information. From the particle size distribution in Figures S3c and S4c in the Supporting Information, the even size of Pt_1_Pd_2_/C and Pt_1_Pd_4_/C catalysts is 2.6 and 2.4 nm, respectively. The Pt–Pd alloy NPs in Figures S3b and S4b in the Supporting Information are also mainly (111) facet. This consistency in TEM images of Pt–Pd/C catalysts indicates that the electron reduction can control the morphology of alloy NPs despite of the changing of the composition and the loading of these alloy NPs. For comparison, Figures S5 and S6 (Supporting Information) show TEM images of commercial Pt/C and Pt_1_/C catalysts. As shown in Figure S5a,c (Supporting Information), the Pt NPs of the commercial Pt/C catalyst dispersed on the carbon display a uniform size of about 2.9 nm. The Pt NPs in the Figure S5b in the Supporting Information can be clearly identified with lattice fringes attributed to (111) and (220) facets. For the Pt_1_/C catalyst shown in Figure S6c in the Supporting Information, the size of Pt NPs decreases to 2.6 nm due to the electron reduction. The Pt NPs in Figure S6b in the Supporting Information also show mainly (111) facet, which is the same as Pt–Pd/C catalysts. This indicates that the electron reduction method could control the size and the facet of NPs effectively, no matter these NPs are single element or alloy. According to EDS spectra in Figures S3d and S4d in the Supporting Information, the Pt/Pd weight ratios of Pt_1_Pd_2_/C and Pt_1_Pd_4_/C catalysts are almost 1:2 and 1:4.1, respectively. Therefore, the Pt loading of Pt_1_Pd_2_/C and Pt_1_Pd_4_/C catalysts are about 5.0% and 2.5%.

**Figure 3:**
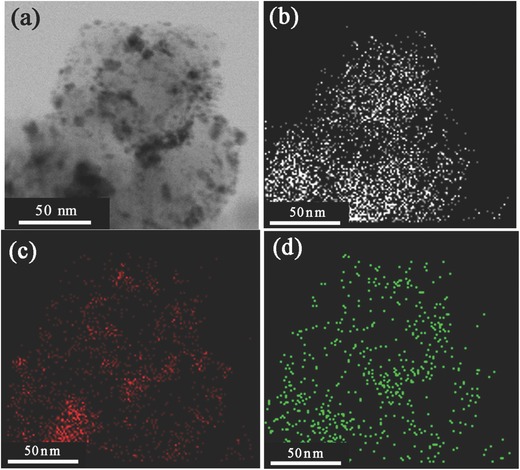
EDS mapping scanning of the Pt_1_Pd_1_/C catalyst. a) TEM image, b–d) the corresponding EDS mapping scanning results: b) C, c) Pd, and d) Pt.

Besides the results of TEM, EDS mapping scanning was also used to confirm the NPs of Pt–Pd/C catalysts being in alloy form. The EDS mapping scanning images of Pt_1_Pd_1_/C, Pt_1_Pd_2_/C, and Pt_1_Pd_4_/C catalysts are shown in **Figure** **3**, and Figures S7 and S8 in the Supporting Information, respectively. From Figure 3, it can be found that both Pt and Pd are distributed throughout the carbon support, especially for the region on which more NPs dispersed. This is a strong evidence for the formation of Pt–Pd alloy. For Pt_1_Pd_2_/C and Pt_1_Pd_4_/C catalysts in Figures S7 and S8 in the Supporting Information, the distributions of Pt and Pd are almost the same, except that the amount of Pt is lower as the Pt loading decreases. This suggests the formation of Pt–Pd alloy for all Pt–Pd catalysts, though the composition of alloy NPs changed. However, as the size of Pt–Pd alloy NPs is too small to take the EDS line scanning and the carbon support moves continuously, it is hard to determine the structure of Pt–Pd alloy NPs only through TEM results.

**Figure 4:**
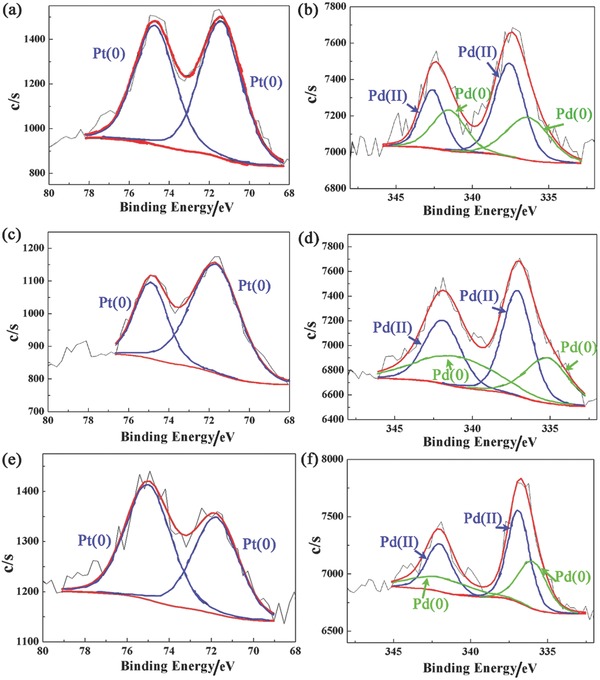
XPS spectra of Pt 4f orbitals and Pd 3d orbitals of Pt–Pd/C catalysts. a,b) Pt_1_Pd_1_/C catalyst, c,d) Pt_1_Pd_2_/C catalyst, and e,f) Pt_1_Pd_4_/C catalyst.

Since the TEM results and EDS mapping scanning only show the existence of Pt–Pd alloy, X‐ray photoelectron spectroscopy (XPS) and X‐ray diffraction (XRD) were used to analyze the structure of Pt–Pd alloy NPs. From XPS results, the composition and the electronic structure of surface and near surface regions could be examined. Figure S9 (Supporting Information) shows the XPS spectra of Pt–Pd/C catalysts. For XPS spectra of Pt_1_Pd_1_/C, Pt_1_Pd_2_/C, and Pt_1_Pd_4_/C catalysts shown in Figure S9 (Supporting Information), the mass ratios of Pt:Pd near the surface are 2:1, 2:1.5, and 2:3, respectively. The high mass fraction of Pt indicates that Pt atoms are concentrated on the surface as the shell. As the Pt loading decreases, the thickness of Pt shell becomes thinner and the mass fraction of Pd increases consequently. The XPS spectra of Pt 4f and Pd 3d of Pt–Pd/C catalysts are shown in **Figure** **4**. For the Pt_1_Pd_1_/C catalyst, the binding energies of Pt^0^ 4f (4f_7/2_ = 71.49 eV; 4f_5/2_ = 74.87 eV) and Pd^0^ 3d (3d_5/2_ = 336.33 eV; 3d_3/2_ = 341.53 eV) are slightly different from the standard values of bulk Pt (4f_7/2_ = 71.30 eV; 4f_5/2_ = 74.50 eV) and bulk Pd (3d_5/2_ = 335.20 eV; 3d_3/2_ = 341.10 eV). These slight differences of peak locations originate from the electronic interactions between the Pt shell and Pd core.^21^ In addition, due to the low loading and high dispersion of Pt, there is slight difference in the area ratios of 4f 7/2 and 4f 5/2 peaks of different catalysts. However, for Pd element, the area ratios between two orbital peaks are almost the same, as the Pd loading is constant for different catalysts. Besides it, the Pd 3d orbitals also show the existence of Pd^2+^. This is because the surface of Pd is easily oxidized at room temperature, especially during the drying process.^16^ It is clearly observed that, for Pt–Pd/C catalysts, the peak intensity of Pd 3d and Pd^2+^ are both weaker as the Pt loading increases. This tendency also shows the existence of Pt shell. Because of the increasing of Pt loading, a continuous, thick, and protective Pt shell is formed. Then, the Pd 3d peaks are suppressed by Pt shell and the Pd core would be more likely to remain in the metallic state.^16^ Therefore, the peak intensities of Pd 3d and Pd^2+^ are both weaker. From these results of the XPS spectra, it can be found that the Pt–Pd alloy NPs in Pt–Pd/C catalysts are core–shell structure. This conclusion is also clarified by XRD patterns.

**Figure 5:**
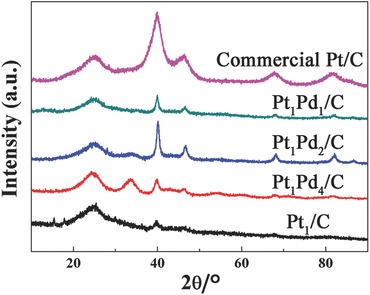
XRD patterns of all catalysts.

The XRD patterns of all catalysts are presented in **Figure** **5**. For the commercial Pt/C catalyst, peaks appear at 2θ = 39.76°, 46.24°, 67.45°, and 81.29°, which are assigned to the (111), (200), (220), and (311) reflections of the face‐centered cubic (fcc) Pt crystalline lattice (PDF#65‐2868). A broad peak for amorphous carbon also exists in the patterns. For Pt–Pd/C catalysts, the XRD patterns also show the fcc structure. Compared with the commercial Pt/C catalyst, the diffraction peaks are slightly higher shifted to 2θ = 39.98°, 46.62°, 68.02°, and 82.29°, suggesting the alloy formation between Pt and Pd, which is in agreement with results of EDS mapping scanning.^22^ For Pt–Pd/C catalysts, besides the peaks of Pt–Pd alloy, there is also a peak existing at 2θ = 34.54°, which corresponds to PdO. As the Pt loading increases, this peak is much weaker and even disappears for the Pt_1_Pd_1_/C catalyst, which is consistent with the conclusion of XPS spectra that Pt atoms are really concentrated on the surface as the shell. And the thickness of Pt shell would be thicker due to the increasing of the Pt loading, so that the peak of PdO becomes much weaker.

**Scheme 1:**
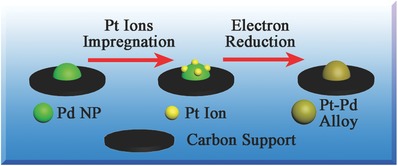
Schematic illustration showing the forming process of Pt–Pd core–shell alloy structure.

According to our previous studies[[qv: 19a,20a]] and theoretic studies by Neyts and co‐workers,^23^ when additional metal precursor ions are introduced to metal‐support material between two electron reduction processes, these ions will tend to adsorb on the metal nanoparticles instead of the support due to the lower surface energy. After the second electron reduction, these additional ions will be reduced to form the alloy or aggregates with the original metal nanoparticles.[[qv: 19a,20a]] In this work, when preparing Pt–Pd/C catalysts, Pd ions were first loaded on the carbon support and reduced to nanoparticles. Then, Pt ions were introduced to the obtained sample and adsorbed on the surface of Pd NPs. At last, Pt ions were reduced on the surface of Pd NPs as a shell and form the alloy nanoparticles with the existing Pd NPs as a result. From the results discussed above and also from our previous works, the conclusion can be made that Pt and Pd in Pt–Pd/C catalysts are formed as core–shell alloy structure. The core and the shell of these alloy NPs are Pd and Pt, respectively. And the thickness of Pt shell would be thicker with the increasing Pt loading (**Scheme** **1**).

### 2.2 Electrocatalytic Tests

**Figure 6:**
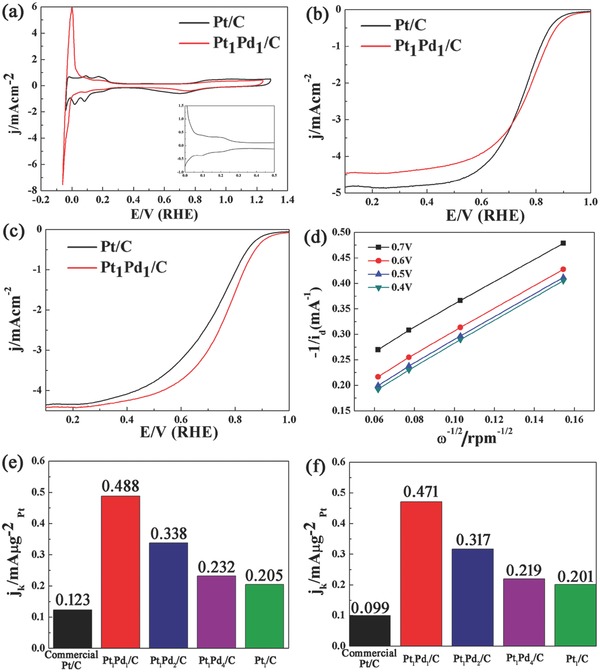
The results of electrocatalytic tests of all catalysts. a–c) Comparison of commercial Pt/C and Pt_1_Pd_1_/C catalysts: a) CV curves in an N_2_‐purged 0.5 m H_2_SO_4_ solution with a sweep rate of 50 mV s^−1^ (inset: the enlarged hydrogen adsorption potential region of the Pt_1_Pd_1_/C catalyst), ORR polarization curves of two catalysts b) before and c) after the accelerated durability test in an O_2_‐saturated 0.5 m H_2_SO_4_ solution with a sweep rate of 10 mV s^−1^ and a rotation rate of 1600 rpm, d) Koutecky–Levich plots of the Pt_1_Pd_1_/C catalyst, and MA results of all catalysts e) before and f) after the accelerated durability test at 0.85 V versus RHE.

The electrocatalytic properties of all catalysts were measured by the rotating disk electrode (RDE) technique. **Figure** **6** shows the results of electrocatalytic tests for all catalysts, including cyclic voltammetry (CV) curves (a), ORR polarization curves (b) before and (c) after the accelerated durability test of commercial Pt/C and Pt_1_Pd_1_/C catalysts, Koutecky–Levich plots of the Pt_1_Pd_1_/C catalyst (d), and MA values of all catalysts at 0.85 V (e) before and (f) after the accelerated durability test. The CV and ORR polarization curves of Pt_1_Pd_2_/C, Pt_1_Pd_4_/C, and Pt_1_/C catalysts are shown in Figures S10–S12 in the Supporting Information, respectively. All potentials in these figures are converted to against the reversible hydrogen electrode (RHE). The CV curves of two catalysts (Figure 6a) exhibit three distinctive potential regions. The most important one is between 0 and 0.35 V, which is attributed to the adsorption/desorption processes (H^+^ + e^−^ = H_upd_) of the underpotentially deposited hydrogen (H_upd_). This region is called the hydrogen adsorption potential region. Another one is the double‐layer potential region (between 0.35 and 0.5 V), which is followed by the oxide formation potential region (beyond 0.5 V).^24^ Besides, the CV curves show clearly crystal structure of Pt NPs of two catalysts. For the commercial Pt/C catalyst, the peaks at 0.08 V are assigned to the (110) sites and that at 0.13 V to the step contributions associated with (100)/(111) terrace sites.^25^ However, for the Pt_1_Pd_1_/C catalyst, the peak attributed to (110) becomes much weaker while that associated with (100)/(111) terrace sites changes slightly. This suggests that the amount of (110) decreases significantly than that of (100)/(111) sites, which is in consistence with results of TEM images. This phenomenon is also found in the CV curve of the Pt_1_/C catalyst in Figure S12a (Supporting Information). For Pt_1_Pd_2_/C and Pt_1_Pd_4_/C catalysts, due to the low Pt loadings, the peaks of Pt facets in their CV curves are not evident (Figures S10a and S11a, Supporting Information). In addition, by measuring the charge collected in the H_upd_ adsorption/desorption region after double‐layer correction and assuming a value of 210 mC cm^−2^ for the adsorption of an hydrogen monolayer, the electrochemically active surface areas (ECSA) of two catalysts were calculated.^26^ The ECSA of the commercial Pt/C catalyst is 63.6 m^2^ g^−1^ while that of the Pt_1_Pd_1_/C catalyst is 107.7 m^2^ g^−1^. The significant improvement of ECSA indicates the great electrocatalytic characteristic of the Pt_1_Pd_1_/C catalyst.

The ORR polarization curves for commercial Pt/C and Pt_1_Pd_1_/C catalysts are shown in Figure 6b at 1600 rpm. For both the catalysts shown in Figure 6b, the diffusion‐limiting currents were obtained in the potential region below 0.4 V, while a mixed kinetic‐diffusion control region occurs between 0.6 and 1.05 V. The onset potential of ORR curves of two catalysts are 1.0 and 1.05 V, respectively. The positive shift of the onset potential indicates the better electrocatalytic activity of Pt_1_Pd_1_/C intuitively.^27^ The kinetic current *j*_k_ was then calculated using the observed current density (*j*) and diffusion‐limited current density (*j*_D_) at 0.85 V. According to the calculations, *j*_k_ of the Pt_1_Pd_1_/C catalyst is 0.976 mA cm^−2^, 1.7 times greater than that of the commercial Pt/C catalyst (0.567 mA cm^−2^). Besides, the transferred electron number of the Pt_1_Pd_1_/C catalyst was also calculated by using the experimental‐derived *B*‐factor from Koutecky–Levich plots (as shown in Figure 6d). The result of calculation is 3.8, which is nearly to that of pure sputtered Pt, suggesting the four electron reduction kinetic of ORR.[[qv: 25a]] For a better understanding of the observed differences in ORR activity, the kinetic current is normalized against the Pt mass (MA) of each catalyst. Figure 6e shows the values of MA of all catalysts. The Pt_1_Pd_1_/C catalyst has the largest MA of 0.488 mA µg^−1^, which is four times greater than that of the commercial Pt/C catalyst (0.123 mA µg^−1^), revealing the significant improvement of ORR activity by forming Pt–Pd core–shell structure through an electron reduction method. Compared with previously reported ORR activities of other Pt–Pd/C catalysts (Table S1, Supporting Information), the onset potential and MA values of our Pt–Pd/C catalyst are higher. For Pt_1_Pd_2_/C and Pt_1_Pd_4_/C catalysts (Figure 6e; Figures S10b and S11b, Supporting Information), the MAs are 0.338 and 0.232 mA µg^−1^, which are 2.7 and 1.8 times greater than that of the commercial Pt/C catalyst, respectively. Though the MAs of Pt–Pd/C catalysts reduced, which originate from the discontinuous and thin Pt shell as the Pt loading decreasing, the Pt–Pd/C catalysts still show the brilliant ORR activity.[[qv: 15a]] In addition, the Pt_1_/C catalyst shows the MA of 0.205 mA µg^−1^ (Figure 6e; Figure S12b, Supporting Information), which is larger than that of the commercial Pt/C catalyst, but smaller than that of Pt–Pd/C catalysts. This comparison suggests that the electron reduction method also has a contribution to the high ORR activity of Pt–Pd/C catalysts. In this study, the ORR activities of the commercial Pt/C catalyst are in good agreement with the reported or predicted values in literature.^28^

**Scheme 2:**
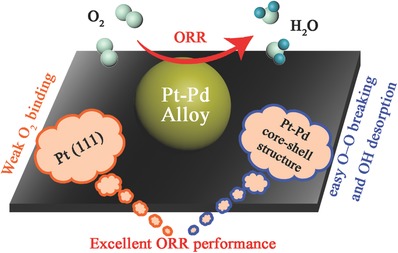
Schematic illustration depicting the main features responsible for the enhanced electrocatalytic performance of ORR over the Pt–Pd/C catalyst.

According to the density functional theory (DFT) and experimental study of Shao et al.,^29^ the O—O binding is the weakest for all sites of Pt NPs with diameter of 2.2 nm, and the binding on (111) facet is significantly weaker than other facets. The weaker O—O binding could facilitate the breaking of O—O bonds, which benefits the ORR performance of the Pt catalyst as a result. For Pt–Pd/C and Pt_1_/C catalysts, the size of Pt NPs is about 2.6 nm and the surface sites feature mainly (111) facet. This small size and the change of facet mean that the O—O bindings on Pt NPs of Pt–Pd/C and Pt_1_/C catalysts are much weaker than that of the commercial Pt/C catalyst. That is why the Pt_1_/C catalyst also shows better ORR activity than that of the commercial Pt/C catalyst. Therefore, the ORR activity of the Pt–Pd/C catalyst is increased due to the electron reduction treatment. Besides it, as the Pt–Pd alloy NPs forming the core–shell structure with Pt as the shell, the addition of Pd plays a very important role in the high ORR activity of Pt–Pd/C catalysts. It is well known that the ORR rate is controlled by two steps: the breaking of O—O bands and desorption of an OH.^30^ Many DFT and experimental studies have indicated that both two steps could be promoted by forming the Pt–Pd core–shell structure.^31^ This is because the energy of d‐band center of Pt could be decreased with the existence of Pd, which is called the “ligand‐effect.”^32^ On one hand, according to the DFT studies, because of the lower d‐band center energy, more 5d vacancy of Pt will exist and the interaction between O_2_ and Pt will be increased with 0.3 eV as a result, which facilitates the electron transfer and the O—O bond cleavage.^33^ On the other hand, the bond energy between adsorbed OH and the Pt shell will be lower due to the downshift of the Pt d‐band center with respect to the Fermi level, which has been confirmed by DFT and experimental studies.[[qv: 12a,31b]] So that, the amount of OH blocked on the Pt shell could be significantly decreased and ORR activity is consequently enhanced. Therefore, by improving the breaking of O–O bands and desorption of OH, the ORR activity of Pt–Pd/C catalysts is much enhanced (**Scheme** **2**).

Accelerated durability tests for all catalysts were also performed by applying linear potential sweeps between 0.5 and 1.0 V at 50 mV s^−1^ in N_2_‐saturated 0.5 m H_2_SO_4_ solution. Then, ORR polarization curves were recorded at a rotating rate of 1600 rpm after the potential cycles in the durability test (Figure 6c) and values of MA of all catalysts after 10 000 cycles are shown in Figure 6f. After 10 000 cycles, the Pt_1_Pd_1_/C catalyst shows an MA loss of only 3.5% (0.471 mA µg^−1^), whereas the commercial Pt/C catalyst shows a loss of 19.5% in MA (0.099 mA µg^−1^). Due to the significant reduction of MA losses, the MA of the Pt_1_Pd_1_/C catalyst after 10 000 cycles increases to 4.7 times greater than that of the commercial Pt/C catalyst. Compared with previously reported ORR stabilities of other Pt–Pd/C catalysts (Table S1, Supporting Information), our Pt–Pd/C catalyst shows the lowest MA loss. This is because, compared with other fabrication methods, the electron reduction method significantly enhances the interaction between Pt–Pd alloy NPs and carbon support, which will effectively avoid the catalyst poisoning during the ORR.^34^ Such enhanced interaction has been observed in our previous studies in electrocatalysis,[[qv: 18b]] photocatalysis,[[qv: 34a]] and heterogeneous catalysis.[[qv: 34b]] For Pt_1_Pd_2_/C and Pt_1_Pd_4_/C catalysts (Figure 6f; Figures S10c and S11c, Supporting Information), the losses of MA are only 6.2% and 5.6%, respectively. The results of the accelerated durability tests indicate that Pt–Pd/C catalysts have remarkably enhanced stabilities by forming Pt–Pd core–shell structure through the electron reduction method.

## 3 Conclusion

In summary, highly active and stable Pt–Pd/C alloy catalysts have been synthesized by the room‐temperature electron reduction method. The as‐prepared Pt–Pd alloy NPs show a particle size of about 2.6 nm and feature mainly (111) facet, which makes the oxygen binding much weaker. On the other hand, the Pt–Pd alloy NPs are formed in the core–shell structure with Pt as the shell. Both the breaking of O—O bands and desorption of OH are promoted by this structure. The MA of Pt_1_Pd_1_/C catalysts is about four times greater than that of the commercial Pt/C catalyst. The Pt–Pd/C catalyst is very stable, as demonstrated by the 96.5% retention of the mass activity after 10 000 cycles standard durability test. This stability is also much higher than that of the commercial Pt/C catalyst. These findings demonstrate that the Pt–Pd/C alloy catalysts serve as a promising candidate for ORR in both activity and stability.

## 4 Experimental Section

*Materials*: All chemical materials used were obtained commercially and used without further purification. The carbon black support (Vulcan XC‐72) was purchased from Cabot Corp. Palladium nitrate (99.8% with metal basis) was purchased from Alfa Aesar (Tianjin, China). Chloroplatinic acid (99.0% in purity) was purchased from Tianjin Jinbolan Fine Chemical Plant (Tianjin, China). The commercial Pt/C catalyst (Pt loading 20.0%) was purchased from Alfa Aesar (Tianjin, China).

*Catalyst Preparation*: Two steps of wetness impregnations were employed to prepare a series of Pt–Pd alloy catalysts with different Pt–Pd mass ratios. The carbon black support was first impregnated with an aqueous solution of Pd(NO_3_)_2_ (0.1 m) for about 12 h. The obtained sample was dried at 110 °C for 12 h and then treated by the glow discharge electron reduction for 60 min. The details of the electron reduction have been reported in the previous work.^18^ Then, the treated sample was impregnated with an aqueous solution of H_2_PtCl_6_ (0.1 m) for about 12 h again. After the same drying and electron reduction processes, the obtained catalyst was denoted as Pt_1_Pd_1_/C as the feeding composition of two metals were both 10%. Other catalysts with different Pt–Pd mass ratios were denoted as Pt_1_Pd_2_/C (Pt 5%, Pd 10%) and Pt_1_Pd_4_/C (Pt 2.5%, Pd 10%). The commercial Pt/C catalyst was used as the reference sample. For the comparison purpose, another catalyst was also made: the carbon‐black‐supported Pt catalyst by the room‐temperature electron reduction without peptide. This catalyst is denoted as Pt_1_/C. The Pt loading of the Pt_1_/C catalyst is 10%.

*Characterization*: TGA analysis of the catalysts was carried out under a mixed gas atmosphere (total flow: 25 mL min^−1^, O_2_:N_2_ = 1:4) at a constant rate of 10 °C min^−1^, using a Netzsch STA 449 F3 system. The catalyst (≈10 mg) was loaded into an alumina crucible and heated from room temperature to 1000 °C. The XRD patterns of the catalysts were recorded on a Rigaku D/Max‐2500 diffractometer at a scanning speed of 4° min^−1^ over the 2θ range of 10°–90°. The diffractometer was equipped with a Ni filtered Cu Kα radiation source (λ = 1.54056 Å). The phase identification was made by comparison to the Joint Committee on Powder Diffraction Standards (JCPDSs). XPS analyses were performed with a Perkin‐Elmer PHI‐1600 spectrometer using Mg Kα (*hν* = 1486.6 eV) radiation. The binding energy values were calibrated using the C1s peak (*hν* = 284.6 eV) as reference. High resolution transmission electron microscope measurements were performed on a Philips Tecnai G2F20 system equipped with an EDS to determine bulk atomic concentrations, operated at 200 kV. To do so, the catalyst was suspended into the ethanol and dispersed ultrasonically for 30 min. A drop of the suspension was deposited on a copper grid coated with carbon.

*Electrocatalytic Tests*: Electrocatalytic tests were carried out in a conventional three electrodes cell using CHI 660C workstation (CH Instruments) at room temperature. A glassy carbon (GC) disk with a diameter of 5.0 mm was used to prepare the working electrode. First, 1 mg of catalyst was dispersed in 0.5 mL aqueous solution of 0.5% Nafion ultrasonically for 30 min to obtain a homogeneous suspension solution. Then, 5.0 µL of this solution (≈20 µg catalyst) was uniformly pipetted onto the surface of the GC electrode. The modified electrode was used for testing their electrocatalytic features after the coating was dried at room temperature in air. For all catalysts, the platinum plate was used as the counter electrode. The electrolyte was 0.5 m H_2_SO_4_ solution and the Hg/Hg_2_SO_4_ electrode was used as the reference electrode. All potentials are converted to against the RHE in this paper. Before the electrocatalytic tests, the electrode was placed in electrolyte with N_2_ saturated for 10 min and scanned in the potential region from 0.05 to 1.3 V for several cycles at a scan rate of 50 mV s^−1^ until a stable curve was obtained. The CV curves were then recorded with the same condition. The polarization curves for ORR were conducted through the RDE technique at a scan rate of 10 mV s^−1^ with varying rotating speed from 400 to 2500 rpm. The durability tests were conducted with the same conditions in O_2_‐saturated electrolyte for 1600 rpm. Before durability tests, the electrodes were scanned in N_2_‐saturated electrolyte between 0.5 and 1.0 V at a rate of 50 mV s^−1^ for 10 000 cycles. All electrocatalytic tests data analysis is based on the equations reported in our previous work.[[qv: 18b]]

## Supporting information

As a service to our authors and readers, this journal provides supporting information supplied by the authors. Such materials are peer reviewed and may be re‐organized for online delivery, but are not copy‐edited or typeset. Technical support issues arising from supporting information (other than missing files) should be addressed to the authors.

Supplementary
