# Supplementary material for: Highly Active and Stable Pt–Pd Alloy Catalysts Synthesized by Room‐Temperature Electron Reduction for Oxygen Reduction Reaction
Source: Adv Sci (Weinh). 2017 Jan 20;4(4):1600486. doi: 10.1002/advs.201600486 (PMC5396164; doi:10.1002/advs.201600486)
Supplement: Supplementary file 1 — Supplementary [file ADVS-4-na-s001.pdf]

Copyright WILEY-VCH Verlag GmbH & Co. KGaA, 69469 Weinheim, Germany, 2017.

Supporting Information

for *Adv. Sci.,* DOI: 10.1002/advs.201600486

Highly Active and Stable Pt–Pd Alloy Catalysts Synthesized by Room-Temperature Electron Reduction for Oxygen Reduction Reaction

*Wei Wang, Zongyuan Wang, Jiajun Wang, Chuan-Jian Zhong, and Chang-Jun Liu**

Copyright WILEY-VCH Verlag GmbH & Co. KGaA, 69469 Weinheim, Germany, 2016.

Supporting Information

Highly active and stable Pt-Pd alloy catalysts synthesized by room temperature electron reduction for oxygen reduction reaction

*Wei Wang, Zongyuan Wang, Jiajun Wang, Chuan-Jian Zhong and Chang-Jun Liu**

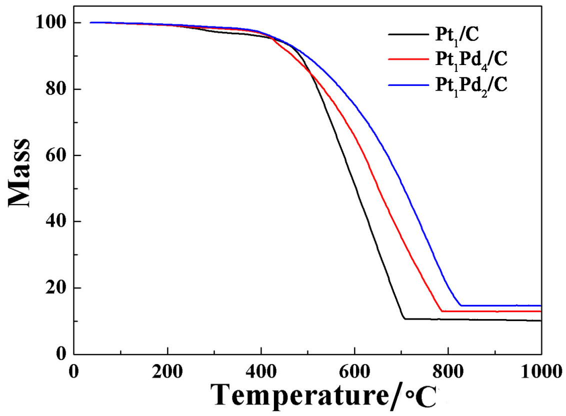

Figure S1. TGA curves of Pt_1/C, Pt_1Pd_2/C and Pt_1Pd_4/C catalysts.

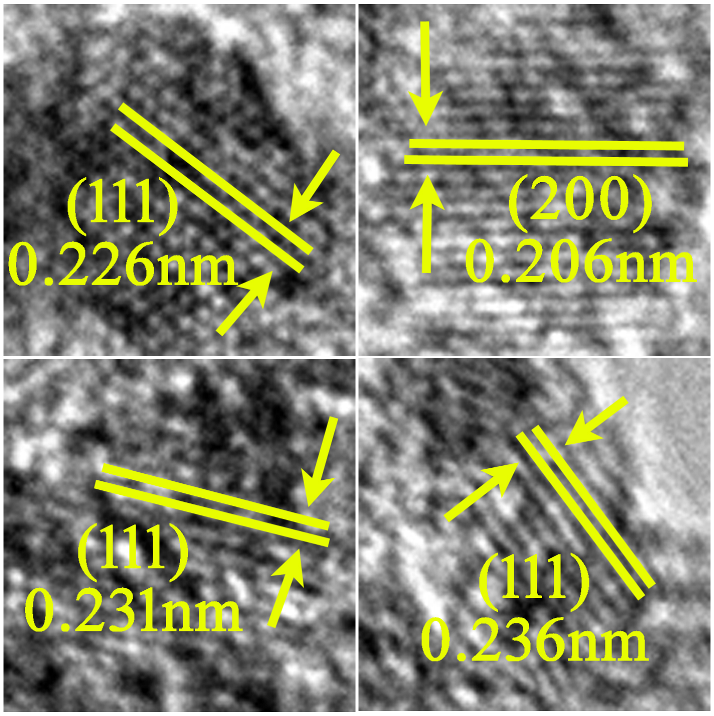

Figure S2. HR-TEM images with clear lattice fringes. These nanoparticles are enlarged with the same nanoparticles shown in Figure 2b.

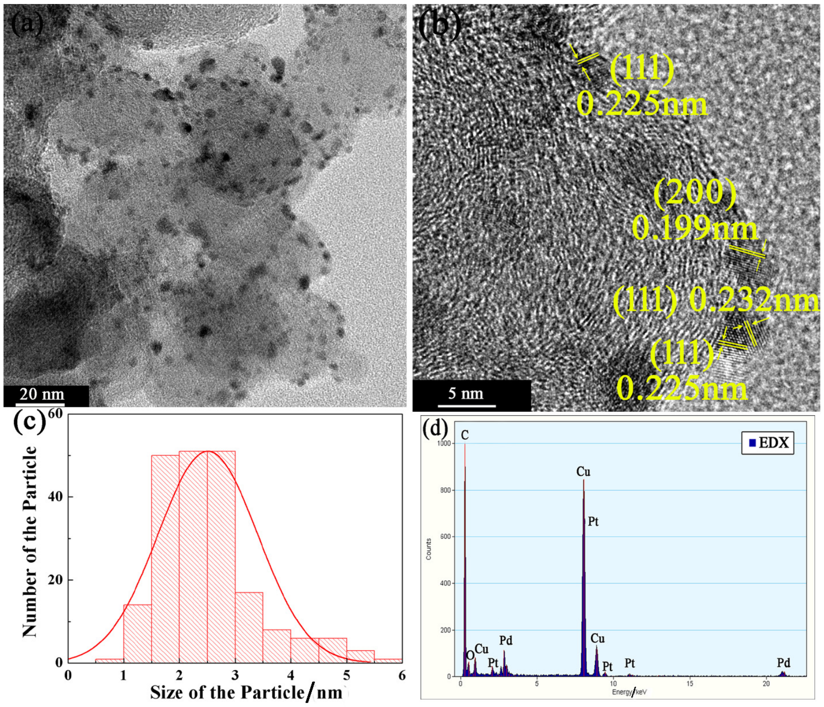

Figure S3. TEM results of Pt_1Pd_2/C catalyst. (a-b) TEM images, (c) the corresponding particle size distribution and (d) EDS spectrum.

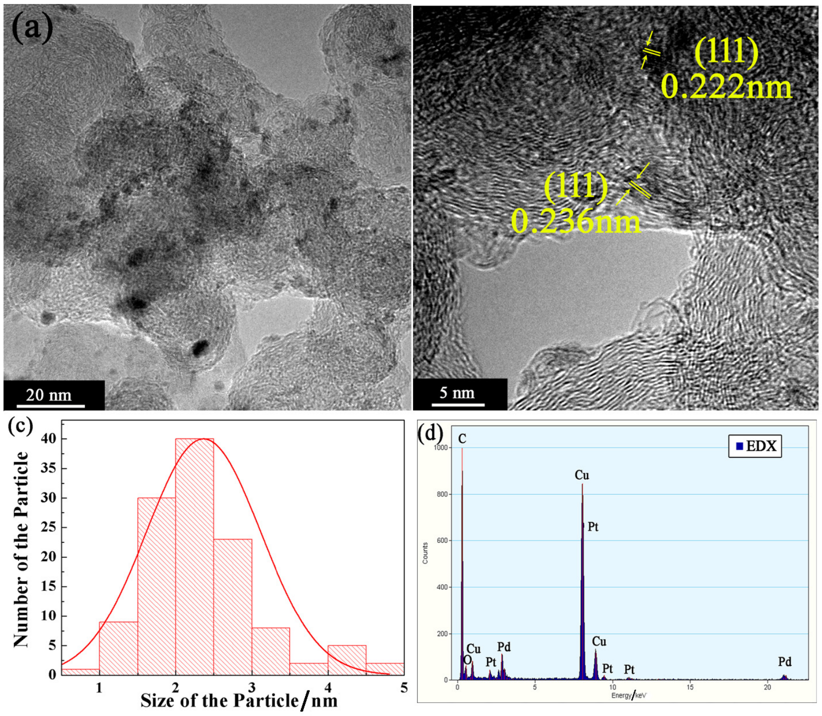

Figure S4. TEM results of Pt_1Pd_4/C catalyst. (a-b) TEM images, (c) the corresponding particle size distribution and (d) EDS spectrum.

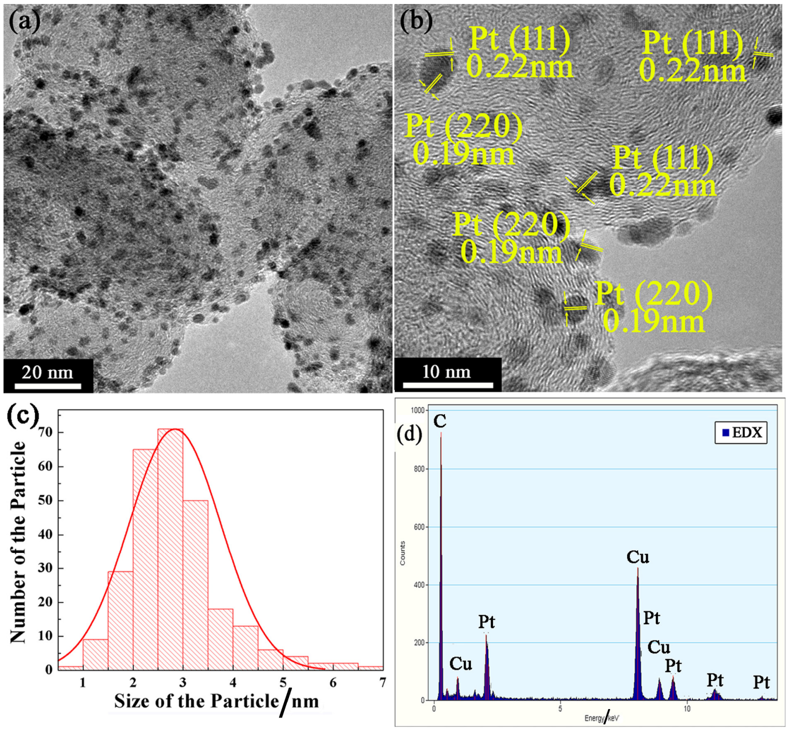

Figure S5. TEM results of commercial Pt/C catalyst. (a-b) TEM images, (c) the corresponding particle size distribution and (d) EDS spectrum.

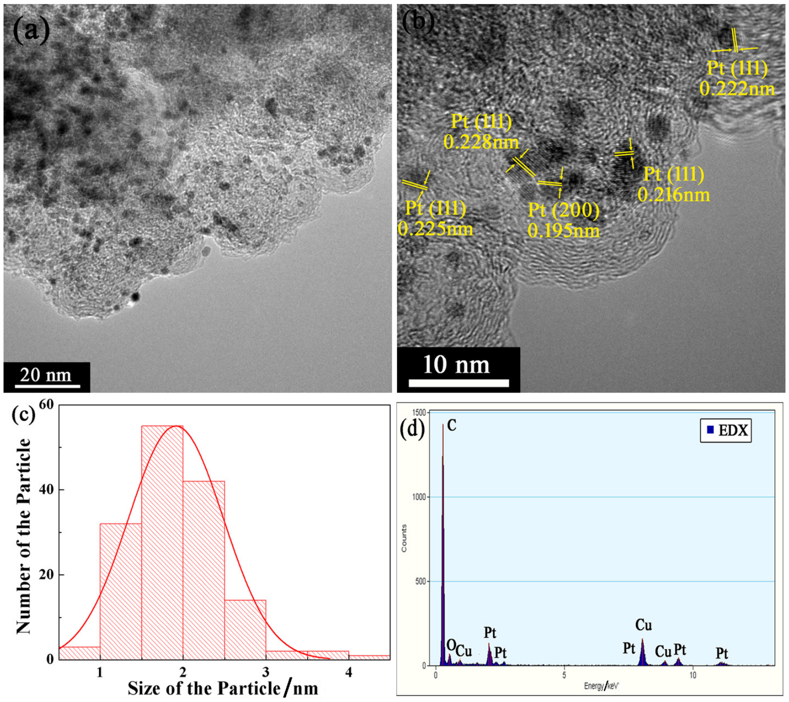

Figure S6. TEM results of Pt_1/C catalyst. (a-b) TEM images, (c) the corresponding particle size distribution and (d) EDS spectrum.

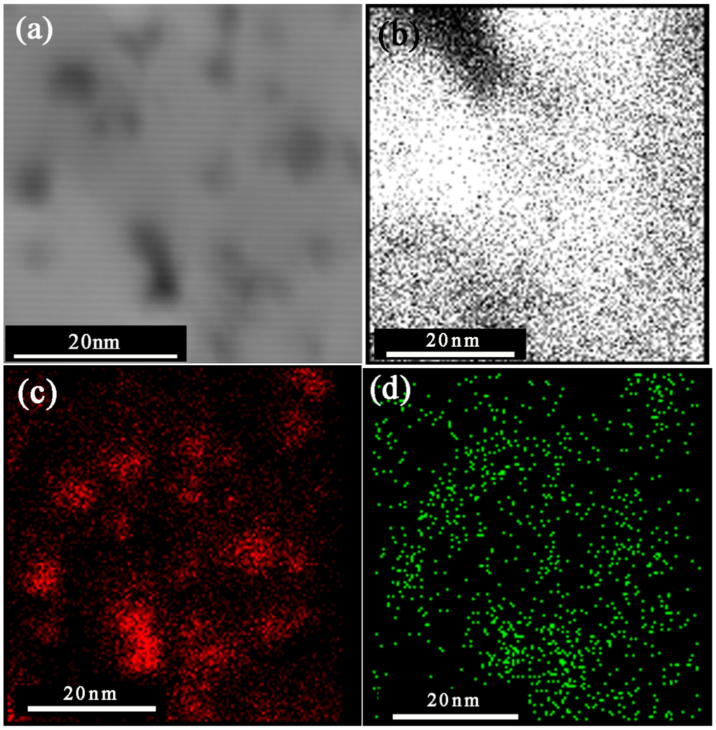

Figure S7. EDS mapping scanning of Pt_1Pd_2/C catalyst. (a) TEM image, (b-d) the corresponding EDS mapping scanning results: (b) C, (c) Pd and (d) Pt.

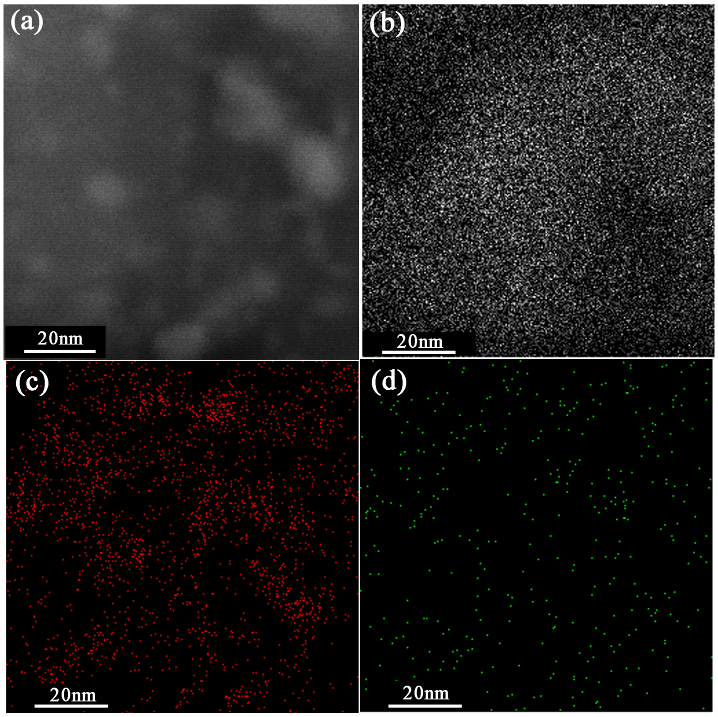

Figure S8. EDS mapping scanning of Pt_1Pd_4/C catalyst. (a) TEM image, (b-d) the corresponding EDS mapping scanning results: (b) C, (c) Pd and (d) Pt.

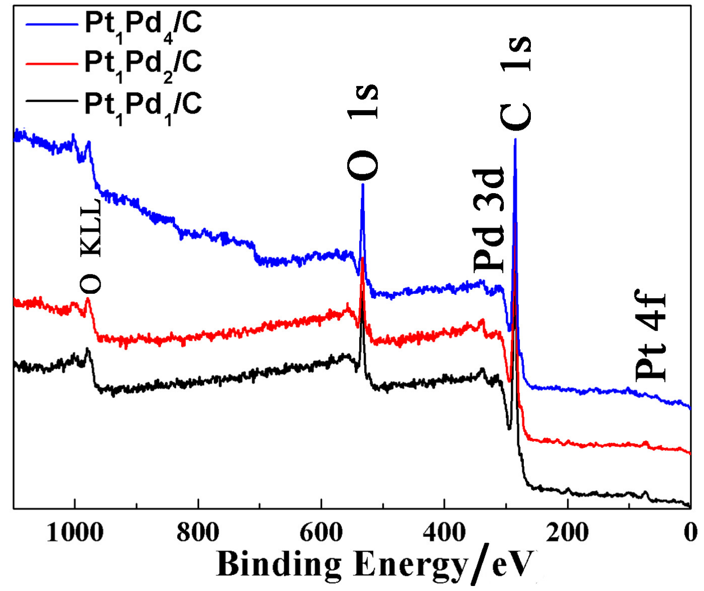

Figure S9. XPS spectra of Pt-Pd/C catalysts.

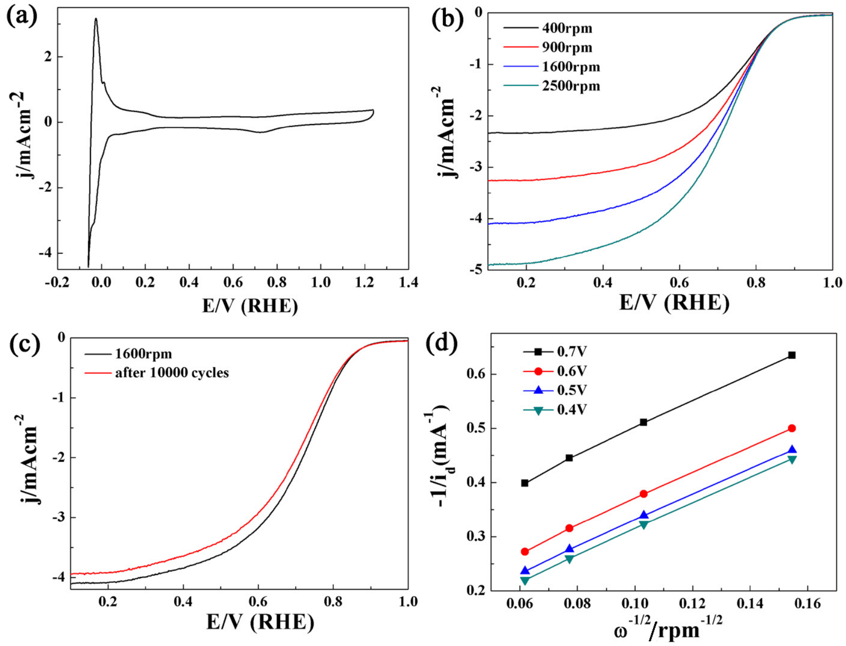

Figure S10. (a) CV curves recorded in an N_2-purged 0.5 M H_2SO_4 solution with a sweep rate of 50 mV s^{-1}, (b) ORR polarization curves with varying rotating speed in an O_2-saturated 0.5 M H_2SO_4 solution with a sweep rate of 10 mV s^{-1}, (c) ORR polarization curves before and after accelerated durability test with 1600 rpm and (d) Koutecky-Levich plots of Pt_1Pd_2/C catalyst.

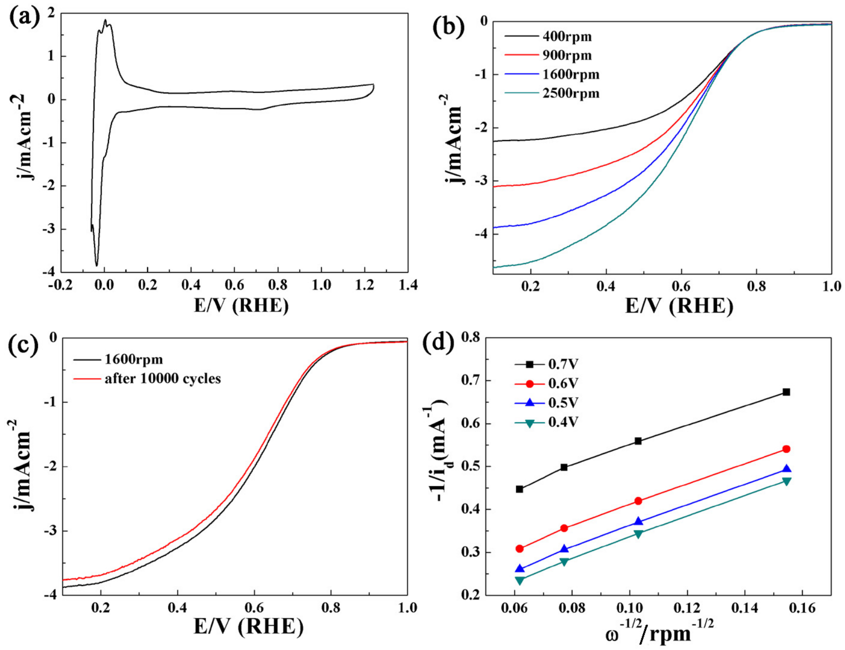

Figure S11. (a) CV curves recorded in an N_2-purged 0.5 M H_2SO_4 solution with a sweep rate of 50 mV s^{-1}, (b) ORR polarization curves with varying rotating speed in an O_2-saturated 0.5 M H_2SO_4 solution with a sweep rate of 10 mV s^{-1}, (c) ORR polarization curves before and after accelerated durability test with 1600 rpm and (d) Koutecky-Levich plots of Pt_1Pd_4/C catalyst.

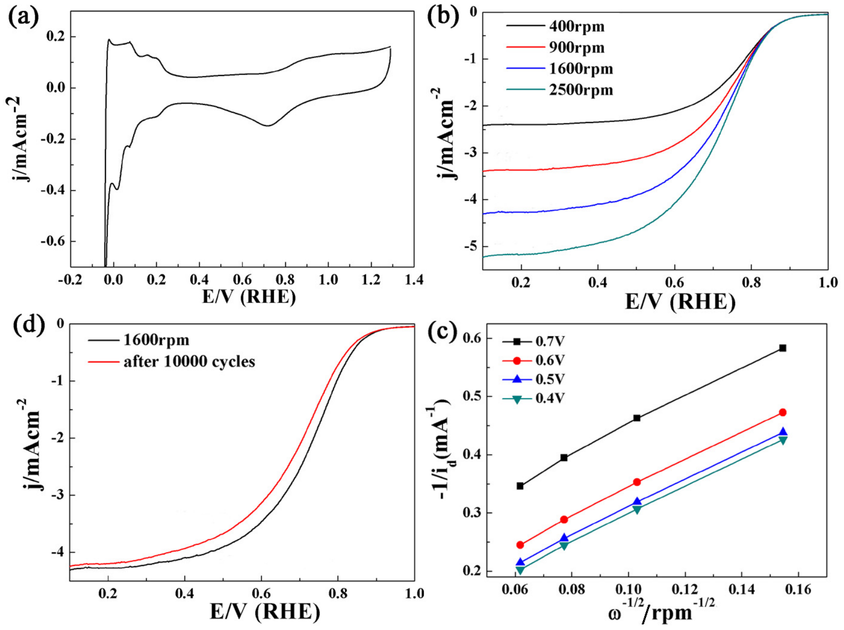

Figure S12. (a) CV curves recorded in an N_2-purged 0.5 M H_2SO_4 solution with a sweep rate of 50 mV s^{-1}, (b) ORR polarization curves with varying rotating speed in an O_2-saturated 0.5 M H_2SO_4 solution with a sweep rate of 10 mV s^{-1}, (c) ORR polarization curves before and after accelerated durability test with 1600 rpm and (d) Koutecky-Levich plots of Pt_1/C catalyst.

Table S1. Comparison of Pt–Pd ORR electrocatalysts

Reference	Synthesis methods and structure	Onset potential (V/RHE)	MA compared to commercial Pt/C catalyst	Remaining percent of MA after durability test
**This work**	**Room temperature electron reduction**	**1.05**	**4 times**	**96.5 %**
S1	Reducing K_2PtCl_4 with L−ascorbic acid with Pd nanocrystal seeds existing in solution	1.0	2.1times	50 %
S2	Pumping Na_2PtCl_6 solution into the reaction solution with Pd icosahedra, KBr, PVP, AA and EG	1.05	7.8 times	60.3 %
S3	Injecting Na_2PtCl_6 solution into the solution with Pd decahedral seeds, KBr, PVP, AA and EG	1.05	5 times	43.1 %
S4	Stepwise Pt−monolayer depositions on Pd/C	1.05	5.6 times	NA
S5	Pt−Pd hydrogels were prepared via the mixing reaction of K_2PdCl_4/H_2PtCl_6 with $NaBH_4$	NA	4.7 times	88 %
S6	Synthesizing Cu nanowire firstly, then adding $PtCl_2$, $PdCl_2$ and DMSO solution dropwise to form PtPdCu alloy nanoparticle nanotubes	1.0	5.3 times	88.3 %
S7	A solution–phase method for the conformal deposition	1.0	3 times	88 %
S8	Adding K_2PtCl_4 and citric acid solution into a suspension consisting of Pd/C nanoparticles	1.0	4 times	82 %
S9	Chemical reduction method using sodium borohydride	0.95	1.32 times	58.7 %

References

[S1] B. Lim, M. Jiang, P. H. C. Camargo, E. C. Cho, J. Tao, X. Lu, Y. Zhu, Y. Xia, *Science*, **2009**, *324*, 1302.

[S2] X. Wang, S. -I. Choi, L. T. Roling, M. Luo, C. Ma, L. Zhang, M. Chi, J. Liu, Z. Xie, J. A. Herron, M. Mavrikakis, Y. Xia, *Nat. Commun*. **2015**, *6*, 7594.

[S3] X. Wang, M. Vara, M. Luo, H. Huang, A. Ruditskiy, J. Park, S. Bao, J. Liu, J. Howe, M. Chi, Z. Xie, Y. Xia, *J. Am. Chem. Soc*. **2015**, *137*, 15036.

[S4] J. X. Wang, H. Inada, L. Wu, Y. Zhu, Y. Choi, P. Liu, W. -P. Zhou, R. R. Adzic, *J. Am. Chem. Soc*. **2009**, *131*, 17298.

[S5] W. Liu, P. Rodriguez, L. Borchardt, A. Foelske, J. Yuan, A. K. Herrmann, D. Geiger, Z. Zheng, S. Kaskel, N. Gaponik, R. Kötz, T. J. Schmidt, A. Eychmüller, *Angew. Chem. Int. Ed*. **2013**, *52*, 9849.

[S6] H. -H. Li, C. -H. Cui, S. Zhao, H. -B. Yao, M. -R. Gao, F. -J. Fan, S. -H. Yu, *Adv. Energy Mater*. **2012**, *2*, 1182.

[S7] S. Xie, S. -I. Choi, N. Lu, L. T. Roling, J. A. Herron, L. Zhang, J. Park, J. Wang, M. J. Kim, Z. Xie, M. Mavrikakis, Y. Xia, *Nano Lett*. **2014**, *14*, 3570.

[S8] L. Zhang, S. Zhu, Q. Chang, D. Su, J. Yue, Z. Du, M. Shao, *ACS Catal*. **2016**, *6*, 3428.

[S9] S. -Y. Huang, P. Ganesan, B. N. Popov, *ACS Catal*. **2012**, *2*, 825.
